# A new “practical" plane for Eustachian tube measurements and its application in predicting middle ear dysfunction in patient with acquired cholesteatomas

**DOI:** 10.1007/s00405-022-07345-3

**Published:** 2022-03-31

**Authors:** Dominic Ku, Bridget Copson, Mark Fiorentino, Jean-Marc Gerrard, Stephen O’Leary

**Affiliations:** 1 Department of Otolaryngology, The Royal Victorian Eye and Ear Hospital, 32 Gisborne St, East Melbourne, VIC 3002 Australia; 2 Department of Radiology, St Vincent’s Hospital, Melbourne, Australia; 3 Department of Radiology, John Hunter Hospital, Newcastle, Australia; 4 University of Melbourne, Melbourne, Australia

**Keywords:** Otology, Radiology, Middle ear dysfunction, Eustachian tube, Auditory tube, Cholesteatoma

## Abstract

**Setting:**

The Eustachian tube plays a vital role in middle ear physiology. There has been evidence that Eustachian tube (ET) and angle are correlated with middle ear function. The measurements of these Eustachian tube features are now made possible with computed tomography and multiplanar reconstruction techniques. However, there has not been a standardised protocol devised to these measurements in limited window cone-beam CT scans of temporal bones.

**Objective:**

The primary object of the present study is to establish and validate a new landmark in closer proximity to the middle ear that is consistently captured, thereby allowing ET angle and length to be measured from the majority of cone-beam CT scans. Secondarily, the ET anatomies of patients with middle ear dysfunction manifesting as acquired cholesteatoma are analysed with this new method of measurement.

**Methods:**

This study undertook a step-by-step method to first validate the methods of ET measurement with Reid’s standard plane, then identifying an alternative landmark, thus a new plane visible on limited window cone-beam CT scans of temporal bones and lastly, validating the application of this new plane in the measurements of ET angle and length. This new method of measurement was coined the Ku-Copson plane and was applied to 30 cochlear implant patients and 30 patients with acquired cholesteatomas. Their ET anatomies were analysed and compared.

**Results:**

It was found that the new Ku-Copson mandibular fossa plane was a reliable and accurate plane for the measurement of ET angle and length. Furthermore, it was found that patients with acquired cholesteatomas have statistically significant smaller ET angles and shorter ET lengths when compared with patients with cochlear implants, of normal middle ear function.

**Conclusion:**

The newly proposed method utilising the right mandibular fossa as an anatomical landmark for ET angles and lengths measurement appears to be viable. The close proximity of this landmark to the middle ear means that it is highly likely to be captured in most cone-beam CT scans of the petrous temporal bones. This enables the retrospective examination ET angles and lengths to be conducted on CB CT scans. This study reports statistically significant difference in ET anatomy in patients with middle ear dysfunction.

## Introduction

The Eustachian tube (ET) plays a vital role in maintaining normal middle ear physiology through two-directional functions [1]. The ET equalises pressure in the middle ear to that of atmospheric pressure by passing small gas boluses in the direction necessary. It protects the middle ear from retrograde migration of microorganisms, reflux of secretions, clears contaminants from the middle ear and functional sounds of the oral cavity and epipharynx. The shape and orientation of the ET are thought to have functional implications on middle ear disease, however the evidence on this topic is inconclusive. In children, the literature is divided on the correlation between ET length and susceptibility to otitis media with effusion (OME) [2, 3]. Though in an adult population, shorter and more horizontal ETs are correlated with chronic suppurative otitis media (CSOM) and cholesteatoma [3]. With the improvement in imaging technologies, it is pertinent that the method of assessment of the ET dimensions is also reassessed.

The increased availability of high-resolution temporal bone computed tomography (CT) and the use of cone-beam CT, combined with the advent of multiplanar reconstruction (MPR) has made it possible for ETs to be measured on imaging studies [1–3, 10, 11]. A review of the literature revealed a range of methods in measuring ET angle and lengths, as summarised in Table 1. There were commonalities in the different methods in the definition of anatomical landmarks, these were largely consistent with what was defined by Takasaki et al. [2]. The pharyngeal orifice of the ET was defined as the nearest point in the pharynx where the loop-shaped taurus tubarius appears. The tympanic orifice of the ET was defined as the nearest point in the ET before the external auditory canal appears on axial images. The estimated length of the ET was defined as the distance between the two points. The studies differed in establishing the plane, the line of the ET is measured against in calculating the angle of the ET. Takasaki et al. [2] used the Reid’s standard plane, which was defined as the plane connecting the right infraorbital margin and the upper margins of the external auditory canals. Dinç et al. [3] used the Frankfort plane as a reference point, which was defined as the left infraorbital margin and the upper margins of the external auditory canals. Other authors only measured the length of the ET and did not measure the ET angle [1, 11].

**Table 1:** Summary of Eustachian tube measurements in the literature

Author	Imaging modality	Anatomy measured	Method
Takasaki 2007 [2]	CT temporal bone (CT PTB)	ET length and angle	The length of the ET was defined as the distance from the pharyngeal orifice to the tympanic orifice (definitions in main text)
			Angle of the ET was defined as the angle of a straight line representing the length of the ET against Reid’s standard plane

Dinç 2015 [3]	CT PTB	ET length and angle	Used basal turns of cochlears as reference point (black arrows) and Frankfort line to adjust AP axis. Landmarks as defined in Takasaki et al

Falkenberg-Jensen 2018 [1]	CT PTB	Cartilaginous portion of ET length	Coronal reformats of 2 points on a single CT image were made, projecting 1) cranial limit (bony eminence separating carotid canal from bony ET) and caudal limit (tip of the soft tissue lip of the torus tubarius)

Ha 2019 [7]	Cone beam CT and CT PTB	Patulous ET length	Multiple points on the ET were traced on consecutive axial CT slides from the nasopharynx to the bony ET The images were reconstructed by the axis of the two points and the distance measured

A commonality among the above-mentioned studies was the use of the infraorbital margin as an anatomical landmark to establish a plane by which the ET angle can be measured against. At our centre of practice, the restricted windows in cone-beam CT temporal bone scans do not capture these landmarks frequently. The present study aims to establish and validate a new landmark in closer proximity to the middle ear that is consistently captured, thereby allowing ET angle and length to be measured from the majority of cone-beam CT scans. This will allow morphological ET study to be standardised in the future. We hypothesized that the mandibular fossa may be a reliable anatomical landmark to achieve this purpose.

## Methodology

The aim of this study is to devise a new method of Eustachian tube length and angle measurement that is suitable for cone-beam CT scans of petrous temporal bones, as there is often a limit to the anterior extent of the scan excluding the conventional landmarks of Reid’s standard plane and Frankfort plane. This study utilised cone-beam CT sinus scans and cone-beam CT petrous temporal bone scans. Cone beam CT sinus scans (NewTom 5G) were obtained with focal spot size of 0.15 mm, kVP 110, 15–30mAs and slice thickness of 0.3 mm. Cone beam CT scans of temporal bones (NewTom 5G) were obtained with focal spot size of 0.15 mm, kVP 110, 15–30 mAs and slice thickness of 0.15 mm.

This study is divided into three parts, and a validation of the results by an independent Clinical Radiology Registrar. The sample size calculation was based on the measurements of Takasaki et al. [2], with 95% confidence, 80% power and the largest reported standard deviation in the ET angle measurements of 4.8°, the minimum number of ears required is twenty four. This study involved a total of sixty patients (120 ears), divided into two cohorts: (1) 30 adult patients with CT sinus scans (16 males, 14 females; age range 18–71 year, mean age 46 year) and (2) 30 adult patients with cone-beam CT temporal bone scans (17 males, 13 females; age range 19–84, mean age 60 year).

### Part one: ET measurement with Reid’s standard plane

The first step in our methodology was to measure ET angle and length with a pre-existing method. Retrospective analysis of 30 randomly selected cone-beam sinus CT scans with no sinus, rhinopharynx or middle ear cleft pathology identified from 2018 to 2019 was conducted. The patients selected were adult patients from the hospital’s rhinology surgery list and did not have a history of middle ear diseases. Analysis was conducted with the multiplanar reconstruction function of syngo.via (©Siemens Healthcare GmbH 2009–2018). The method we adopted was described by Takasaki et al. [2], featuring the Reid’s standard plane as a plane of reference. This was defined as a plane connecting the right infraorbital margin and the upper margins of the external auditory canals (EACs). Next, the pharyngeal orifice of the ET was identified on the axial images (the first slide when the loop-shaped torus tabarius was in view), a marker was placed at this point. Then, the tympanic orifice of the ET was identified on the coronal images (the first slide when the inferior margin of the EAC was in view). The length of the ET is measured on the coronal image showing both pharyngeal and tympanic orifices. The angle of the ET was measured with reference to the Reid’s standard plane. Measurements were made for each ear of all 30 patients.

### Part two: identifying alternative landmark visible in cone-beam CT scans of petrous temporal bones and validating its use

**Fig. 1:**
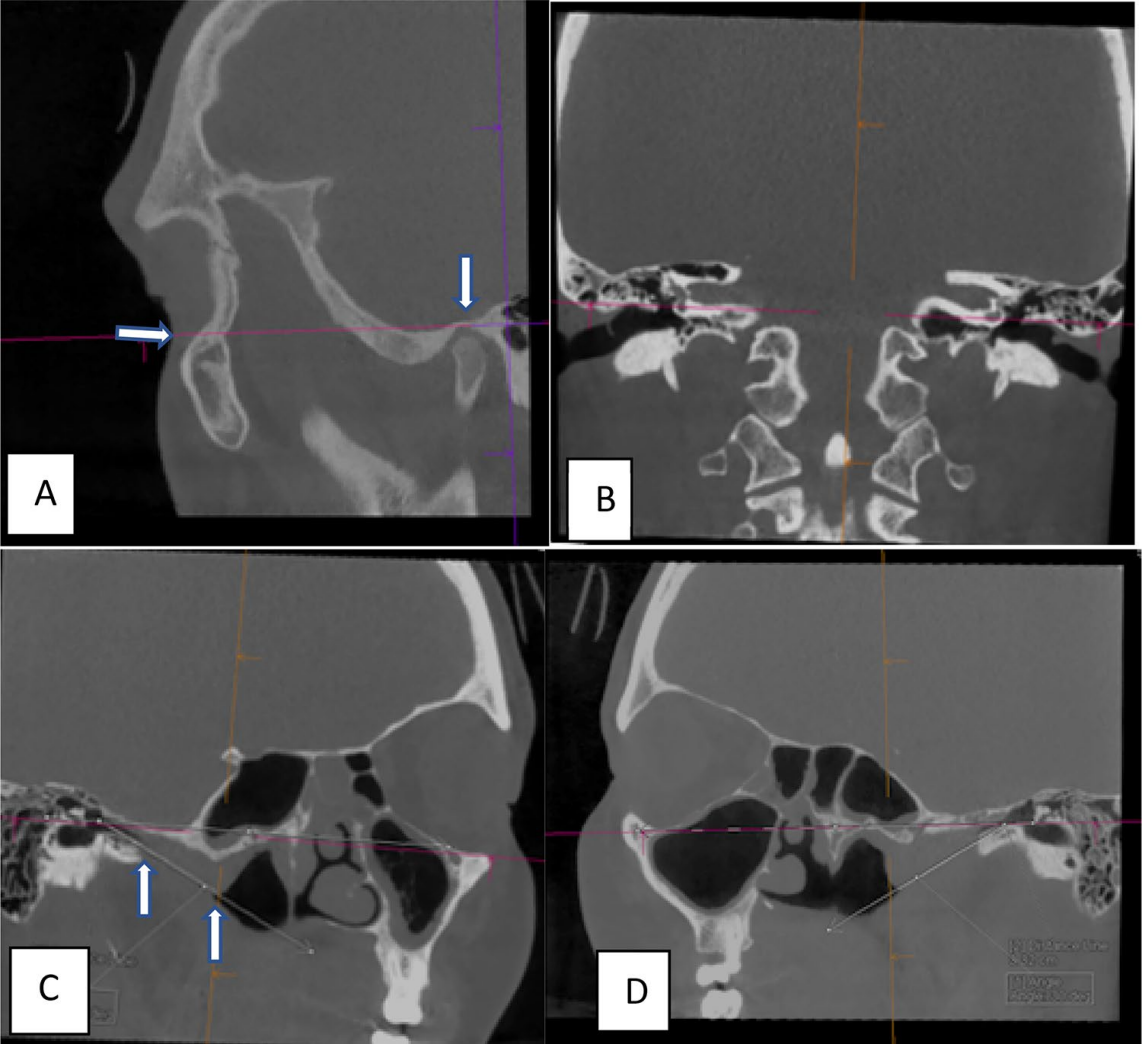
Development of the Ku-Copson plane on MPR analysis of CT PTB scans. **A** The right infraorbital margin (left arrow), right mandibular fossa (right arrow) and the upper margin of the right EAC are on the same plane. **B** The plane is adjusted to course through bilateral upper margins of EACs. **C** The right pharyngeal orifice (right arrow) and tympanic orifice (left arrow) are identified, the distance measured, and ET angle measured with reference to the mandibular fossa plane. **D** The left ET length and angle were measured

The second step was to identify an anatomical landmark closely related to the middle ear, which is captured in cone-beam CT scans of the temporal bone in most cases. The right mandibular fossa was selected for this purpose to replace the right infraorbital margin (See Fig. 1). While the other two landmarks of the Reid’s standard plane were retained, namely bilateral upper margins of the EACs. We coined the new plane the Ku-Copson (KC) mandibular fossa plane. The cone-beam sinus CT scans of the same 30 patients as part one were re-examined. The ET lengths were again measured between pharyngeal and tympanic orifice. The ET angle was measured against the newly established plane involving the right mandibular fossa. The two sets of measurements were statistically compared.

### Part three: validating the results with an independent radiologist

An independent Clinical Radiology Registrar randomly selected ten patients from the cone-beam sinus CT scans cohort by random number generation and analysed these patients’ ET lengths and angles by the two methods: Reid’s standard plane and mandibular fossa plane. These measurements were statistically compared to the primary investigator’s measurements with two-tailed unpaired t test.

### Part four: applying the “Ku-Copson” mandibular fossa plane in cone-beam CT scans

The Ku-Copson (KC) mandibular fossa plane was applied on the cone-beam CT scans of petrous temporal bones in 30 randomly selected adult patients who had undergone cochlear implant surgery in 2018–2019. These patients did not have a history of middle ear disease or surgery. These measurements were statistically compared to the measurements made with MFP in the cone-beam sinus CT scans with two-tailed unpaired *t* test.

### Part five: comparing ET anatomy in patients with cholesteatoma versus patient undergoing cochlear implant as measured by “Ku-Copson” plane

The KC plane was applied to a cohort of the most recent 30 patients aged 18 years and older with middle ear disease in the form of acquired cholesteatoma undergoing surgery before 1 October 2021. The ET anatomy of this cohort was statistically compared to the aforementioned cohort of cochlear implant patients without history of middle ear disease with two-tailed unpaired *t* test. The patient demographics were compared with Fisher’s exact test.

## Results

In Part One of this study, the mean values and standard deviations (SD) of the angles and lengths of right and left ETs as measured on sinus CT scans are shown in Table 2. There was no significant difference between the right and left sides in the 30 adult patients analysed. There was no significant difference between male and female ET lengths.

**Table 2:** ET measurements with reference to Reid’s standard plane on CT sinus scans

Measurements, *n*	Mean (SD)	*p* value
Right ET angle, 30	32.8° (5.6)	0.83
Left ET angle, 30	33.1° (5.1)	
Right ET length, 30	32.5 mm (5.0)	0.84
Left ET length, 30	32.7 mm (5.0)	
Male, 48 vs Female, 12	33.2 (5.1) vs 30.3 (3.1) mm	0.07

In Part Two of this study, the mean values and SD of the angles and lengths of right and left ETs as measured with the Reid’s standard plane were compared to the measurements from the KC mandibular fossa plane and are shown in Table 3. There was no significant difference between the ET measurements.

**Table 3:** Comparison of ET measurements measured with reference to Reid’s standard plane (RSP) vs Ku-Copson plane

Measurements, *n*	Mean (SD)	*p* value
RSP ET angle, 60	33.0° (5.3)	0.07
MFP ET angle, 60	34.7° (5.2)	
RSP ET length, 60	32.6 mm (4.9)	0.10
MFP ET length, 60	31.3 mm (3.7)	
Male, 48		
RSP ET length	33.2 mm (5.1)	0.10
MFP ET length	31.7 mm (3.8)	
Female		
RSP ET length	30.3 mm (3.1)	0.71
MFP ET length	29.8 mm (3.1)	

In Part Three of this study, an independent Clinical Radiology Registrar randomly selected ten patients and conducted ET measurements with reference to both Reid’s standard plane and MFP. These measurements were compared to the measurements made by the primary investigator. There was no significant difference between the ET measurements.

In Part Four of this study, the Ku-Copson mandibular fossa plane was applied to a new cohort of patients on cone-beam CT scans of temporal bones. These patients have had imaging investigations in preparation for cochlear implantation; they did not have a history of middle ear disease and had normal tympanometry. These measurements are shown in Table 4. There was no difference between the measured ET angles and lengths when compared to the sinus CT scan cohort.

**Table 4:** Comparison of ET measurements with reference to Ku-Copson plane in cohort in cone-beam CT (CBCT) vs in cohort with sinus CT scans

Measurements, *n*	Mean (SD)	*p* value
CBCT ET angle, 60	33.0° (6.1)	0.11
Sinus CT ET angle, 60	34.7° (5.2)	
CBCT ET length, 60	31.3 mm (3.7)	0.26
Sinus CT ET length, 60	31.9 mm (2.4)	

In Part Five of this study, a comparison of the patient demographics revealed a significantly older cochlear implant cohort compared to the cholesteatoma cohort (63.9 vs 42.5 years, *p* = 0.0001). There was no significant difference in gender distribution or place of birth as shown in Table 5.

**Table 5:** Comparison of patient demographics in cohort with cochlear implant (CI) vs in cohort with cholesteatoma (CH)

Patient demographics	Mean (SD)/*n* (%)	*p* value
Age in years		
Cochlear implant	63.9 (21.3)	0.0001
Cholesteatoma	42.5 (14.3)	
Number of females		
Cochlear implant	14 (46.7%)	0.43
Cholesteatoma	10 (33.3%)	
Number born in Australia		
Cochlear implant	22 (73.3%)	0.064
Cholesteatoma	14 (46.7%)	

The comparison of ET measurements demonstrated significantly more horizontal ET angles in the cholesteatoma cohort as measured with reference to the Ku-Copson mandibular fossa plane (27.8° vs 33.0°, *p* = 0.0001). The cholesteatoma cohort also had significantly shorter ET lengths (25.9 vs 31.9 mm, *p* = 0.0001) (Table 6).

**Table 6:** Comparison of ET measurements with reference to Ku-Copson plane in cohort with cochlear implant (CI) vs in cohort with cholesteatoma (CH)

Measurements, *n*	Mean (SD)	*p* value
Cochlear implant ET angle, 60	33.0° (6.1)	0.0001
Cholesteatoma ET angle, 60	27.8° (5.1)	
Cochlear implant ET length, 60	31.9 mm (2.4)	0.0001
Cholesteatoma ET length, 60	25.9 mm (5.1)	

## Discussion

The present study builds on the foundations of ET measurements using CT images and multi-planar reconstruction (MPR) techniques [2, 3]. In particular, the Reid’s standard plane and landmarks in Takasaki et al. study have been used to establish the baseline measurements [2]. The Reid’s standard plane is not dissimilar to the Frankfort plane used by Dinç et al. [3] and both required the CT images to capture the infraorbital margins. While cone-beam CT scans of the temporal bones provide great details of the middle ear and inner ear structures, the protocols often do not extend as anterior as sinus CT scans. The orbits are usually excluded from the scan range to practice the “as low as reasonably achievable” (ALARA) technique [12], in particular to protect the radiosensitive lenses. Herein lies the problem that has inspired this study. We would like to accurately measure ET angles and lengths by locating a consistent anatomical landmark within the limited view of CB CT scans.

Part One of this study established the consistency of Takasaki et al.’s method of ET measurement in the present cohort, demonstrating no significant difference between right and left ears, as was reported in the literature [2]. Part Two of this study tested the hypothesis that the right mandibular fossa would be a suitable anatomical landmark to replace the infraorbital margins in the Reid’s standard plane and Frankfort plane. This was validated as there was no significant difference in the measurements obtained with reference to the KC MFP compared to the Reid’s standard plane. The final part of this study was to apply this new method of ET measurement to cone-beam CT scans of temporal bones. We chose the cochlear implant cohort for analysis as they are largely without history of middle ear disease and has a large sample of perioperative CB CT scans available. Again, there was no significant difference in ET measurements in this group compare to the sinus CT scan group. Our results demonstrated that the KC MFP can be used consistently to measure ET angles and lengths in CT scans.

The ET angles in normal adults as measured by Takasaki et al., and Dinç et al., were reported as 27.3 (± 2.8) degrees and 23.6 (± 2.4) degrees respectively. While Proctor’s anatomical report shown adult’s ET angle to be 45°s as measured against the horizontal plane [5]. Our ET angle measurements are in between the values measured by CT MPR and the gross anatomic method of Proctor’s, and closer in value to the CT MPR reports. This validates our results as accurate measurements of ET angles on CT MPR. This is important for future work on this topic, as it is not always practical to measure ET angles with gross anatomic methods. CT MPR measurements are more clinically relevant and more accurate with thinner imaging slices for temporal bone analysis.

ET length is a rather more complex matter. The method used in this study, as well as used in the studies of Takasaki and Dinç et al.’s estimates the true ET length by pharyngeal orifice to tympanic orifice distance. The ET lengths in normal adults were calculated to be 42.7(± 2.9) mm and 39.3 mm (range 31–44) by the aforementioned authors respectively. In the anatomic study with 3D reconstruction by Ishijima et al. [6], it has been shown that the true ET length (ET lumen path length) is longer than that estimated by pharyngeal orifice to tympanic orifice distance. The ET lumen path length can be measured from cadaveric specimens, which were finely sectioned and 3D reconstructed to account for the curvature of the ET at the bony cartilaginous junction [6]. The measurement of ET lumen path length is not possible on CT scans unless under special circumstance such in patients with patulous ET [11], or with invasive injection of contrast media into the middle ear cleft [10].

The present study is a pilot study demonstrating the utility of the Ku-Copson mandibular fossa plane in cone-beam CT scans; as well as establishing baseline ET measurements for a normal adult population. The rationale behind the use of the Ku-Copson mandibular fossa plane is two-fold. First, the apex of the mandibular fossa when compared to the infraorbital margin (in Reid’s plane) can be more reliably measured due to its smaller size, producing less variations between measurements. Secondly, its close proximity to the ET middle ear orifice allows this landmark to be used in both sinus CT scans and cone-beam CT, thereby standardising ET measurements while also minimising unnecessary radiation. The clinical application of the Ku-Copson plane in a cohort of patients with middle ear dysfunction manifesting in acquired cholesteatoma has demonstrated significantly more horizontal Eustachian tube angles and shorter lengths compared to patients with normal middle ear function. This is congruent with published data by Dinç et al. [3]. Clinical application of these findings may contribute to the understanding of pathophysiology and implications in disease prevention.

Future studies may aim to apply this method to a larger population and a wider age group to further validate the method against published data. This study is limited by the retrospective nature of data collection; the Eustachian tube measurements of the cochlear implant cohort and cholesteatoma cohort were conducted separately, and blinding was difficult as cholesteatoma was visible on the images of all patients. Future studies may benefit from prospectively collected randomised data to minimise bias.

## Conclusion

The newly proposed method utilising the right mandibular fossa as an anatomical landmark for ET angles and lengths measurement appears to be viable. The close proximity of this landmark to the middle ear means that it is highly likely to be captured in most cone-beam CT scans of the petrous temporal bones. This enables the retrospective examination ET angles and lengths to be conducted on CB CT scans. Preliminary application of the newly proposed Ku-Copson plane in the clinical context of middle ear dysfunction has demonstrated data congruent with published literature.
